# Ethical standards for mental health and psychosocial support research in emergencies: review of literature and current debates

**DOI:** 10.1186/s12992-017-0231-y

**Published:** 2017-02-08

**Authors:** Anna Chiumento, Atif Rahman, Lucy Frith, Leslie Snider, Wietse A. Tol

**Affiliations:** 1 University of Liverpool, Institute of Psychology, Health and Society, 2nd Floor, Block B, Waterhouse Building, 1-5 Brownlow Street, Liverpool, L69 3GL UK; 2 Independent Consultant, Peace in Practice, Amsterdam, The Netherlands; 3 Johns Hopkins University, School of Public Health and The Peter C. Alderman Foundation, Baltimore, USA

**Keywords:** Research ethics, Mental health and psychosocial support (MHPSS), Emergencies, Monitoring and evaluation, Conflict, Disaster, Research guidelines, Multidisciplinary literature review, Ethical practice

## Abstract

**Background:**

Research in emergencies is needed to understand the prevalence of mental health and psychosocial problems and strengthen the evidence base for interventions. All research - including operational needs assessments, programme monitoring and evaluation, and formal academic research - must be conducted ethically. While there is broad consensus on fundamental principles codified in research ethics guidelines, these do not address the ethical specificities of conducting mental health and psychosocial support (MHPSS) research with adults in emergencies. To address this gap, this paper presents a review of multidisciplinary literature to identify specific ethical principles applicable to MHPSS research in emergencies.

**Discussion:**

Fifty-nine sources meeting the literature review inclusion criteria were analysed following a thematic synthesis approach. There was consensus on the relevance of universal ethical research principles to MHPSS research in emergencies, including norms of participant informed consent and protection; ensuring benefit arises from research participation; researcher neutrality, accountability, and safety; and the duty to ensure research is well designed and accounts for contextual factors in emergency settings.

We go onto discuss unresolved issues by highlighting six current debates relating to the application of ethics in emergency settings: (1) what constitutes fair benefits?; (2) how should informed consent be operationalised?; (3) is there a role for decision making capacity assessments?; (4) how do risk management approaches impact upon the construction of ethical research?; (5) how can ethical reflection best be achieved?, and (6) are ethical review boards sufficiently representative and equipped to judge the ethical and scientific merit of emergency MHPSS research? Underlying these debates is a systemic tension between procedural ethics and ethics in practice.

**Summary and recommendations:**

In summary, underpinning the literature is a desire to ensure the protection of participants exposed to emergencies and in need of evidence-based MHPSS. However, there is a lack of agreement on how to contextualise guidelines and procedures to effectively maximise the perspectives of researchers, participants and ethical review boards. This is a tension that the field must address to strengthen ethical MHPSS research in emergencies.

## Background

In emergencies - including disasters triggered by natural events and armed conflicts, and associated refugee or internally displaced persons settings - the prevalence of mental health and psychosocial problems is high. Research in emergencies may be aimed at understanding the causes of mental health and psychosocial problems, or the acceptability and effectiveness of mental health and psychosocial support (MHPSS) interventions; and is needed to strengthen the evidence base for policy and practice [1, 2]. Research includes operational needs assessments, programme monitoring and evaluation, as well as formal academic studies; and may be conducted by a variety of actors including United Nations agencies, governmental and non-governmental organisations, academics, and field practitioners.

All research must be conducted ethically. Research guidelines codify the norms underpinning ethical research practice from a range of disciplinary perspectives including biomedical [3–6] and social sciences [7–9]. Existing guidelines do not directly address the ethical specificities of conducting MHPSS research in emergencies [10, 11].

To address this gap, a multidisciplinary literature review was conducted to identify specific principles applicable to ethical MHPSS research with adults in emergencies. The Inter-Agency Standing Committee Reference Group on Mental Health and Psychosocial Support in Emergencies (IASC-RG) supported this effort through input and critical review, and by publishing a set of recommendations for ethical MHPSS research in emergencies based upon this review [12].

In the discussion we identify the strengths and limitations of the review. We then highlight the distinct features of conducting ethical MHPSS research in emergencies, and identify an underlying debate between those who recommend strengthening procedures, and those calling for more flexibility in applying ethical principles to MHPSS research practice.

## Methods

This literature review aims to integrate and interpret empirical evidence on which ethical principles are applicable to MHPSS research with adults in emergencies.

### Search strategy

The following medicine, social science and medical ethics databases were searched: SCOPUS; Web of Science; ProQuest Humanities and Social Sciences and ProQuest Health Sciences; Cochrane Library; MedLine; PROSPERO; PsycINFO; and the WHO Global Health Library and Regional Database. Key search terms included ethic*, research*, evaluat*, humanitarian, conflict, disaster, mental health, and psychosocial, with appropriate MeSH terms derived for each search engine - most commonly: ethic* AND (research* OR evaluat*) AND (humanitarian OR conflict OR disaster) AND (mental health OR psychosocial).

Additional searches were conducted on practitioner databases including mhpss.net; refworld.org; and alnap.org. These provide the humanitarian community with platforms for sharing resources related to emergency MHPSS research, good practice, and policy. Search terms were “ethical” or “ethical research”.

Further literature was identified through cross-referencing citations of included sources and recommendations from the IASC-RG working group supporting this review. Searches were conducted between January and March 2014 by the lead author.

### Inclusion criteria

Literature was considered for inclusion if it discussed ethical considerations relevant to MHPSS research in emergencies, or with refugee or asylum seeking populations. To ensure the practical applicability of findings, “research” was defined broadly covering formal research across academic disciplines, discussion papers, ethical analysis, and operational research such as assessment, monitoring and evaluation of MHPSS programmes. Published and unpublished empirical research and policy guidance were eligible, including reflective researcher and practitioner perspectives. Due to the broad scope of this review, sources were assumed to be of good quality since the majority were drawn from peer-review journals, books, or guidelines likely to have undergone some level of quality assessment.

Other inclusion criteria were publication in English; for academic databases publication in a scholarly peer reviewed journal or book (depending upon the search engine); and full text availability. No geographical or date limiters were set. Literature on research with children was excluded, recognising the additional legal and ethical considerations, principally related to autonomy and capacity. However, the findings from this review similarly apply to research with children, but would require additional ethical assessment.

### Analysis

All sources meeting the inclusion criteria were independently reviewed by the lead author. From this, key data was extracted from each paper on (a) the general ethical principles identified to promote ethical research; (b) the operationalisation of each principle; and (c) commentary on the appropriateness of each principle to humanitarian emergency settings. This extracted data was summarised and shared for review with the IASC-RG working group^1^ comprised of academic researchers, MHPSS practitioners from International Non-Governmental Organisations, the International Federation of Red Cross and Red Crescent Societies, and representatives of United Nations agencies.

Literature was analysed following a thematic synthesis approach [13]. Descriptive themes evolved iteratively alongside the literature review, by identifying and grouping ethical principles according to their role in promoting ethical practice. From this, descriptive themes were mapped to explore possible connections between principles and to identify analytical themes for a practice-focused framework. This process was continued until saturation was achieved. This process was initially conducted by the lead author and refined through monthly discussion with IASC-RG working group members. Once a framework was finalised, the literature was revisited and re-analysed by the lead author to ensure findings remained grounded in the data.

Through this process, key tensions across the literature surrounding the application of ethical principles to emergency MHPSS research practice became apparent. These emerged either as discussion points across papers, or through differing recommendations for managing or resolving key ethical issues. Therefore, in the final section of the paper, we outline six key debates to highlight why and where these controversies arise; offering researcher’s suggested topics to reflect upon their own ethical practice.

## Results

**Fig. 1:**
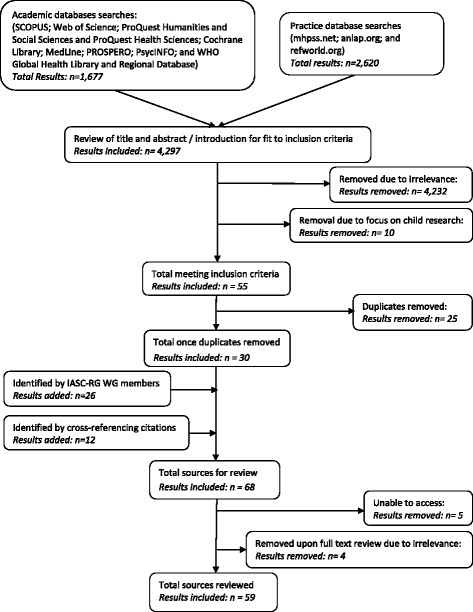
Flow chart of literature searches

Academic and practice database searches retrieved 4297 results (1677 and 2620 results from each database respectively). Of these, review of the title and abstract or introduction led to removal of 4232 papers as not relevant, 10 for focusing upon research with children, and 25 duplicates. Further sources were added by IASC-RG Working Group members (n = 26) and through cross-referencing (n = 12). When conducting full text review five results were removed due to inaccessibility, and four for irrelevance. Therefore, combined searches on academic and practice databases identified a total of 59 results for inclusion in the review (see Fig. 1).

Table 1 presents the results according to five inter-related and overarching ethical principles: (1) Scientific research design; (2) Participation; (3) Safety; (4) Neutrality and (5) Purpose and Benefit. Each one represents the end-result or intended outcome of ethical practice and contains sub-themes of ethical considerations to be addressed. For example, informed consent is viewed as important to achieving the ethical principle of participation.

**Table 1:** Thematic analysis of ethical principles applicable to MHPSS research in emergencies

Ethical principle	Themes	Sub-themes	Source reference papers
Scientific research design	Selection of research question	Necessity	[14, 21, 24–32]
		Researcher inherent biases	[17, 21, 33, 34]
	Risk/benefit evaluation	Emergency = heightened risk	[14, 15, 21, 25, 31, 35, 38–42, 46]
		Benefits relative to burdens	[1, 14, 15, 18, 19, 21, 26, 29, 31, 32, 35–37, 43, 44, 46]
	Appropriate methodology	Lack of methodological rigor	[22, 37, 38, 47, 48]
		Methodological transparency	[17, 18, 21, 24, 28, 30, 35, 45, 49–51]
		Methods implemented well	[17, 21, 23, 26, 28, 29, 31, 32, 35, 40, 43, 52–54]
	Critical reflection	Continuous reflexivity	[26, 29, 33, 43, 52, 55]
		Collective learning	[1, 14, 16, 31, 38, 50, 54]
Participation	Meaningful opportunity for contributing to research design and conduct	Shared understanding	[1, 15, 17, 19–21, 23, 28, 31, 32, 41, 42, 50–52]
		Partnership model	[1, 20, 21, 24, 32, 37, 38, 42, 44, 47, 57]
		Advising on management of ethical issues	[1, 37, 38, 42, 48, 55]
	Fair selection of participants	Selection according to research objectives	[36]
		Risks of targeted selection	[21]
		Informed by local knowledge	[1, 21, 38, 57]
	Informed consent:	Informed consent as an accepted ethical norm	[18, 21, 24, 29, 39, 45, 47, 58, 59]
		As a contested concept	[37, 58, 60]
		As (flexible) process	[14, 21, 38, 44, 51, 52, 57, 60]
		Procedural considerations	[24, 27, 35]
	i. Information provided	Consent as “informed”	[15, 21, 37]
		Information provided	[15, 58, 61]
	ii. Comprehension of information	Strength of information exchange process	[14, 21, 27, 32, 33, 38, 57, 58]
		Barriers to comprehension	[18, 37, 39, 40, 52, 62]
		Strategies to verify comprehension	[14, 32, 37, 62]
	iii. Voluntariness	Factors influencing	[15, 18, 19, 21, 22, 31, 35, 45]
		Potential coercion due to emergency context	[1, 15, 18, 19, 21, 31, 37, 44, 51]
	Autonomy and capacity	Normative connections	[19, 21, 33, 46, 51, 59]
		Decision-making capacity debate	[18, 19, 45, 51, 52]
		Limiting potential exploitation	[1, 31, 38, 54]
		Procedural considerations	[45, 63]
	Confidentiality and anonymity	Increased importance of in emergencies	[14, 28, 31, 35]
		Limits in emergencies	[18, 21, 26, 38, 40, 58, 64]
		Harms if breached	[28, 31, 35, 44, 65]
		Duty to safeguard	[14, 15, 18, 27]
		Management of data	[14, 21, 27, 32, 51]
Safety	Participant vulnerability i. Protection needs	Protection framework	[18, 19, 21, 28, 35, 37]
		Vulnerability: contested concept	[19, 32, 46, 51]
		Individual situational approach	[35, 46]
		Serious mental disorders	[21, 35, 54, 65]
		Potential for exploitation	[21, 36, 45]
	Accountability i. Fair selection and specialist training of research and auxiliary staff	Adequate preparation	[20, 23, 24, 26, 29–32, 42–44, 47, 54, 61, 65]
		Answerable to stakeholders	[47, 54]
		Transparent staff selection	[15, 21, 31, 32, 43, 54–56, 64, 66]
		Specialist training	[1, 14, 21, 23, 24, 27, 32, 35, 39, 43, 45, 51, 52, 54, 55, 58, 61, 65]
		Tensions in collaborative partnerships	[22]
	Researcher self-care	Protecting against negative reactions to emergency context and/or research topic	[1, 14, 21, 23, 24, 31, 34, 35]
		Self- and team-care strategies	[24, 31, 34, 43]
	Environmental, political and health safety	Working “in-extremis”	[28, 35, 43, 61, 63]
		Procedures to respond	[14, 21, 24, 28, 35, 37, 39, 43, 61, 63]
Neutrality	Access and exit strategies i. Gatekeepers and power	Coordinating with existing systems	[20, 23, 24, 61]
		Power & knowledge asymmetries	[21, 22, 28, 32, 35, 64, 67]
		Gatekeepers: benefits and critique of	[15, 20, 21, 28, 33, 35, 51, 56, 64, 68]
		Transparency towards power	[15, 21, 22, 30, 32, 47, 52, 55]
	Coordination with other researchers and organisations	Mutual respect /trust	[38, 66]
		International collaborations and power	[21, 30, 41, 47, 55, 64]
		Networked with emergency response	[20, 26, 29, 31, 43, 52]
		Risk of poor coordination	[20, 21, 52]
	Declaration of researcher interests	Transparency about	[1, 18, 22, 24, 30, 37, 41, 52, 64]
	Funding	Power of	[1, 21, 22, 42, 52]
		Impact of emergency upon budget / funding	[24, 36]
		Advocacy to funders	[1, 21, 22, 56]
Purpose and benefit	Sustainable benefit	Levels of benefits	[1, 21, 24, 35, 37, 38, 41, 42, 49, 68]
		Haphazard process of accruing	[20, 24, 36–38]
		Long-term collaborations & sustainable benefit	[22, 30, 31, 51, 64]
	Dissemination	Right to results	[1, 20, 21, 24, 29, 32, 35, 47, 51, 54]
		Potential risks in	[21, 22, 28, 41, 42, 58]
		Forms of	[1, 14, 24, 30, 63, 67]
		Of data collection tools and methods	[49, 69]
	Ethical review	As accepted norm	[18, 19, 25, 63]
		Responsibilities of reviewers	[22, 24, 37, 38, 46]
		Lack of specificity to emergencies	[1, 21, 22, 31, 39]

Ensuring research is conducted ethically necessitates “a thoughtful process of balancing ethical considerations” [28 - p.936], requiring that researcher’s “bring the question of ethics – too often neglected to a one off aspect of the research process – to something that suffuses all we do” [57 - p.2241]. Ethical themes are presented with an overview paragraph, followed by a brief discussion of sub-themes specific to conducting MHPSS research with populations in emergencies. Whilst themes are presented separately, authors frequently discussed them interdependently, with considerations under one theme typically influencing others.

### Scientific research design

It was generally agreed that ensuring a scientific research design is a core principle of ethical research. Given that much emergency research is conducted in low and middle income countries (LMIC), authors call attention to contextual realities including: culture [14]; patterns and dynamics of conflict [15]; inequity of healthcare [16]; and political and socioeconomic vulnerabilities of individuals and communities [16, 17]. Since emergency research frequently operates alongside relief initiatives, it was stressed that research design should:

- not impede relief [18, 19];
- build upon existing systems and resources [20];
- recognise field practice difficulties, minimising risk [1];
- be conducted at an appropriate time [20]; and
- pay attention to communal and non-pathological processes including resilience, and not only MHPSS vulnerabilities [21–23].

#### Selection of research questions

The findings indicate that research questions require a scientific rationale for *why* the research should be conducted in an emergency, addressing priority unanswered questions [14, 21, 24–26] and not duplicating research [27]. Theoretical and practical relevance should be ensured [28, 29], avoiding over-researching a population [14, 30] including those considered “at risk” [31], and have a purpose beyond contributing to knowledge alone [32].

As in all research, authors caution that all research (including selecting research topics, [21], design, and analysis procedures [17, 33, 34]) must correspond to what emergency-affected communities require or are seeking, and not only be informed by the professional expertise and interests of the researcher. For example, researchers with specialist training – whether in epidemiology, qualitative interviewing, or psychological treatments - should avoid pursuing research questions that align with their strengths and interests where these are not relevant to affected communities’ priorities and needs.

#### Risk and benefit evaluation

Authors stressed the ethical imperative to maximise benefit and minimise harm through a favourable risk/benefit ratio and appropriate strategies to mitigate the inherent risks present in all research [19, 35–37]. This does not require research to be risk free [25], but recognises that emergencies automatically expose participants to higher risks [38, 39].

Whilst what constitutes “fair” benefits was contested, there was broad agreement that benefits be defined in direct relation to burdens: as risks increase, so should the benefits [36]. Certain risk / benefit considerations were identified as requiring special attention in emergencies:

- awareness of socio-political context [1, 15] including safety considerations such as when gathering groups [21];
- strength of confidentiality and anonymity procedures, avoiding (inadvertent) disclosure, recognising the harm this may cause to individuals and communities [21, 35, 40];
- understanding the impact dissemination may have upon communities receiving aid and services [41, 42];
- adequate responses to research participants’ discomfort or adverse reactions, including functional referral pathways for MHPSS care [14, 18, 26, 29, 31, 32, 35, 43, 44] determined by the level of risk that the research [45] or the participant’s situation presents [46];
- communication of risks and benefits in informed consent, identifying risks that matter to participants in/following a particular emergency [39].
- ensuring researcher self-care [14, 31].

#### Appropriate methodology

A number of sources stated that to undermine the research methodology is to undermine its ethical status [22, 37, 38, 47, 48], arguing that ethically no data is better than bad data [21]. An appropriate methodology involves transparency about methods, results, and limitations - including potential sources of bias such as sentimentality [18, 28, 30, 49, 50]. This underscores the idea that to over generalise or promote knowledge founded upon unreliable methods may cause harm [28].

It was advised that protocols clearly outline the research design [17, 21, 28]. Considerations specific to emergencies include making explicit how contextual norms are addressed [35], inform study design, and will be evaluated during the research life-cycle [24]. Also emphasised was an assessment of how informed consent processes respond to changing circumstances [24, 51], and to participants with potentially impaired decision making capacity [45].

Methodologically sound research requires methods to be practiced well [52]. Avoiding labelling, stigmatising or pathologising participants is viewed to be particularly important for populations who may be disempowered following an emergency [21, 31, 35, 43]. To achieve this, researchers need to be aware of contested or culturally rooted concepts such as “childhood” [21], and to avoid reified and simplistic understandings of, for example, “community” [31].

Methodological considerations specific to cross-cultural emergency research include:

(i) Cultural adaptation of standardised mental health instruments:
  Ethnographic methods to inform instrument adaptation are recommended to ensure local applicability [21, 23, 29]. Given resource constraints in emergencies, authors promote developing instruments using local clinical standards [29, 53] and evaluation tools that serve both clinical and research purposes [29].
(ii) Conducting interviews:
  Methodological considerations when interviewing in emergencies include: length and format [35, 54], sampling approach [28, 32, 40], asking the right questions [31, 52] using appropriate language and phrasing [17, 21, 35], and being aware of terminology that may reflect a policy stance or researcher sympathies [32]. Interview questions can inadvertently resemble other official procedures (e.g., history taking for refugee status claims) [32], that may lead to participants “performing” [31]. Longer field time for data collection may reveal inconsistencies in participant narratives [28], avoiding the pitfalls of time-bound “fly-in, fly-out” research [26]. For interviews on sensitive topics, authors recommend having a diversionary questionnaire that asks non-sensitive questions (e.g., basic demographic information) to draw upon should interview conditions become unsafe or privacy interrupted [35].
(iii) Interpreters:
  Hynes [32] notes the importance of researcher–interpreter trust. Others emphasise attention to bias in translation as a result of ethnic, cultural or status differences between interpreters and participants [28, 35], as well as the additional burden upon participants when interviews are conducted with interpreters [35].

#### Critical ethical reflection

Critical ethical reflection supports reflexivity towards researcher power [43] and is suggested as a way to promote ethics as a natural discourse in emergency research [55]. Given the particular ethical challenges that may arise in emergency research, authors recommend conducting ethical reflection [1] to increase transparency and learning [14, 16, 38, 54]. In potentially changing contexts there is consensus that ethical issues be evaluated throughout research: in the inception and design phase [26, 29]; during data collection and analysis, extending to dissemination and post-dissemination [33, 52]. To do this, the researcher’s role is reframed from that of “expert” to “co-learner” [31], and for MHPSS practitioners to shift from “being assessed” to “self-assessment” [50].

### Participation

The findings highlight that participation in research is universally viewed as a basic right [21, 29, 31, 51, 56], interacting with other rights such as respect for autonomy and self-determination [20, 51, 56]. In emergencies, participation was viewed as remedying systemic disempowerment of displaced communities [32], rebalancing the researcher / researched relationship [37] by addressing the question of who is being researched and why [42]. Participatory approaches to conducting research can deliver potential benefits to populations exposed to emergencies, such as:

- a pathway to being heard or regaining dignity [21, 31];
- recapturing a sense of control [31];
- ensuring research responds to local needs, priorities, knowledge [1, 28, 52], and values [51], and respects local knowledge [20];
- engaging with service providers [50];
- enhancing public understanding of research [19];
- providing opportunities for community dialogue and engagement on how to manage ethical issues [48, 55], promoting trust and effective research partnerships [55].

#### Meaningful opportunities for contributing to research design and conduct

It was generally agreed that grounding research in local explanatory models of an emergency [17], helps to build a common understanding between the researchers and the community from the outset [32, 42], and to ensure research meets community needs [20]. Participation was defined as collaborative partnerships with shared responsibility in all research stages [38], requiring mutual commitment to a partnership model founded upon trust [1]. Participation builds upon an individual’s capacity to join or lead studies with affected communities [20, 37, 44, 47] and strengthens local institutions - deemed particularly important in LMIC where institutions may be weak or eroded following an emergency.

It was suggested that protocols propose scenarios for community engagement throughout the research life-cycle in each unique emergency context [21, 24, 37, 42, 57]. This includes engaging community participation to identify research questions [42]; methods; tools; approaches to data analysis and interpretation; dissemination routes and formats [1]; protocol development [37]; and approaches to enhance management of ethical issues [38].

In emergencies the benefits of community participation include informing researchers about community-based practices that may protect psychological and psychosocial health [23], and those that may cause harm [52]. Participation can also help to address potential community suspicions relating to why data is being collected [41], and to counteract a “culture of silence” [21 - p.10] adopted by participants as a strategy to minimise exposure to risk [15].

#### Fair selection of participants

Findings highlight that participants should be selected according to the research objectives [36]. Participation can aid in reaching socially marginalised groups [38] and those likely to self-exclude [21]. Cautions were raised that researchers should be aware that participant selection creates perceptions of who is being heard, and may cause intra-community conflict due to perceived discrimination or social injustice [21].

Community involvement in participant selection is seen as a way to provide researchers with an opportunity to learn of ongoing research, and prevent participants from being involved in multiple studies that my lead to burnout [1, 21, 57]. It also offers opportunities for learning about contextual factors such as family or community coercion to participate, or the potential for incentives to be viewed as coercive [38].

#### Informed consent

There was general consensus that informed consent is central to ethical research [18, 21, 29, 47, 58]. Consent is described as being intimately linked to norms of voluntariness, autonomy, and capacity [21, 39, 45]; a process where research objectives and expectations are established [59], and benefits presented and affirmed by participants [24].

Conversely, some authors contest the concept of informed consent, questioning whom it aims to protect [37, 58, 60]. To address this, there is broad support for emphasising the consent *process* beyond providing forms to be read and signed [14, 60], viewing consent as a partnership between researchers and participants [44] that responds to cultural and social practices [38].

Flexibility in obtaining informed consent was recognised as being necessary across different emergency and cultural contexts. Alternatives to written consent are suggested, such as: oral consent [21, 52, 57]; an interviewer signing a form confirming participant consent; or participants signing a separate form that does not identify the study topic – deemed appropriate for sensitive research [14]. Other suggestions for a flexible approach include consent taken at multiple levels [38] and sources [21, 51] including community, elders or leaders, families, and individuals as appropriate to the setting. Taking this further, Mackenzie et al [51] propose approval of a consent framework which ensures norms such as autonomy and capacity are upheld, but that also provide the researcher with flexibility as to how these are implemented and ensured in practice.

Procedural considerations include processes for documenting or recording consent and managing identifiable personal data [24, 27, 35]. Some authors recommend obtaining consent from research staff (e.g., data collectors, auxiliary staff such as drivers), recognising they undertake these roles in a context of additional risks associated with working in emergencies [17, 24, 33]. This is particularly important when engaging student researchers who may feel compelled to take part as part of their studies [18].

##### Information provided

Consent as “informed” is defined universally as: “an understanding of study purpose, who are the targeted beneficiaries, and the implications of involvement…information is communicated in a form appropriate to the culture, age, and educational level of that individual” [14 - p.s224]. Authors place emphasis upon uncoerced decision-making [37] through clear and consistent explanations of research at all stages [15].

For MHPSS research conducted in emergencies, the information provided to participants is similar to that provided for research in non-emergency settings. Additional recommendations specific to emergencies are to provide information on the purpose of research for communities unfamiliar with this concept [37], and on the limits of the researcher’s role to ensure realistic expectations [15, 61]. A further concern specific to MHPSS research in all settings is avoiding therapeutic misconception [58] by clearly differentiating between therapeutic services and research [57], particularly important in emergency settings where resources can be scarce. Harper [58] builds upon this, suggesting that therapeutic misconception is attributable to a transmission model of information transfer that emphasises only the sending and receiving of information, rather than its explanation.

##### Comprehension of information

Simply providing information is not seen as sufficient for *informed* consent. Rather, information *exchange* beyond the informed consent form is viewed as pivotal to avoiding exploitation [14, 33, 57], helping to ensure that information is fully understood and minimising false perceptions [21]. Cultural, linguistic [52], economic, social status, and other barriers [39, 40, 62] between the researcher and participants are emphasised, highlighting the importance of effective communication [18] and the time, skill and resources this requires [37]. Authors recommended using clear local language and terminology [27, 32] presented in an appropriate format [38]. Partnerships with people who have the cultural and linguistic background to maximise comprehension and minimise misunderstanding is suggested as one route to overcoming communication barriers [18]. Other factors that may affect information comprehension include the communication skills and perceived authority of the person taking consent [62], and the use of technology in communication [39].

Fitzgerald et al [62] cite a lack of practical guidance on ensuring full understanding of study information, and recommend an oral examination with participants to verify understanding. Less formally, the World Health Organisation [14] recommends researchers ask participants to repeat back in their own words their understanding of the research, including the key principles of the right to refuse to participate and confidentiality. This approach offers an opportunity to assess participants’ comprehension and to re-explain or rephrase information as required for each participant [14, 32, 37].

##### Voluntariness (including compensation)

Authors recognise factors in emergencies that influence the voluntariness of consent to include: unequal power relationships [21], fear of outsiders [35], incentives or compensation to populations living in a dependent status [15, 18, 19, 21], and cultural or religious values [22, 45] - including where refusal is seen as contrary to hospitality norms [21, 31] or collectivist cultures [22]. Unequal power relationships may raise expectations of research benefits [15] including access to services [21, 51], money, or aid [31]. This is felt to be influenced by the dependence of populations experiencing emergencies [19].

It is questioned whether participants are truly free to say no to research when it is connected to MHPSS services [19, 44]. O'Mathuna [18] suggests emergencies increase the chance that incentives are coercive, where compensation beyond reimbursement of time and/or expenses can be ethically questionable. Zwi et al [1] argue that to ensure voluntariness research benefits must not act as excessive inducement, and should be distributed in a way that maintains confidentiality and doesn’t worsen conflict within communities. Contrastingly, Benatar [37] argues that incentives cannot constitute coercion, recognising that the structural conditions in many LMICs mean that research participation may provide access to unavailable healthcare that populations have a right to, provided that the benefits of participation continue to outweigh the risks.

##### Autonomy and capacity

As a norm, consent is identified to assume participant autonomy [51]. Authors state that upholding autonomy requires considering the capacity of the participant to provide consent [21] (based upon the principle of respect for persons by accounting for individual situational needs and vulnerabilities [46]); and prioritising protection needs over research [19, 33].

Recent debate has focused upon the extent that exposure to emergencies affects decision-making capacity (DMC) [18, 19, 45, 51, 52]. Underlying this debate is a common view that the researcher has a responsibility to ensure respect for autonomy through uncoerced research participation [59]. Not addressing autonomy and capacity is deemed unethical research practice, and as potentially leading to the exploitation of participants. Emanuel et al [38] identify poverty, cultural and linguistic barriers, and limited understanding of research as increasing the chances of exploitation; particularly where regulatory structures to protect participants are underdeveloped. A participatory approach is recommended to identify those with potentially limited autonomy and capacity [31]. This includes recognising varying conceptualisations of autonomy to minimise coercion [54]. In support of a participatory approach, Zwi et al [1] maintain that failure to acknowledge the capacity of emergency-affected communities to take an active role in research is to undermine the potential for innovative studies.

Rosenstein [45] calls for protocols and training on how to identify and respond to those at risk or with impaired DMC. For research involving participants with severe mental health difficulties, Bhan [63] supports obtaining consent from both the participant and family.

##### Confidentiality and anonymity

Authors identify confidentiality, privacy and anonymity as fundamental research principles [14]. It is accepted by many authors that potential harms resulting from breaches of these principles are heightened in emergencies, for example access to resources or causing stigma and community rejection [28, 31, 35].

Authors acknowledge that emergency contexts present challenges to ensuring privacy, and therefore to maintaining confidentiality [26, 64], including efforts not to inadvertently identify a population sub-group [40]. One example is the disclosure of mental health diagnoses that may leave participants open to stigma and community rejection [65], raising protection concerns that can be difficult to address in emergency settings [44]. Media involvement in dissemination may further increase the chance of accidental disclosure [21, 27].

Despite the challenges, the researcher’s duty to safeguard privacy and confidentiality both during and after research is highlighted [15, 18, 27]: “anyone asking someone to disclose information bears a responsibility to safeguard that information” [18 - p.18]. Recommendations are made for explaining confidentiality procedures to participants from initial contact until the research is disseminated, and to ask participants if these are adequate [35]. It is recommended that explanations include stating that absolute confidentiality cannot be guaranteed [38] by outlining foreseeable limits particular to any given emergency [18].

Procedurally, authors highlight that research protocols should identify how confidentiality and data security will be managed [27], including arrangements relating to interpreters [32]. This includes reporting “off the record” statements [51] and how privacy norms will be met, for example in situations where females require a male chaperone to be present during data collection [21]. It is emphasised that all members of the research team, including auxiliary staff, understand, agree to, and sign confidentiality agreements [14].

When considering dissemination, Allden et al [21] identify the challenges to ensuring participants understand the implications of allowing data to be shared or publicised. Furthermore, Harper [58] asks if researchers should be required to return to participants for permission for each use of data not covered in the original consent, such as for teaching purposes.

### Safety

There is agreement amongst authors that protecting participant and researcher safety is essential in emergencies [19, 21, 28], forming one element of accountability to participants and research staff [26, 29, 31, 43, 61]. Ensuring safety is viewed as requiring accountability in staff selection and training [1, 14, 21, 23, 27, 39, 43, 51, 52, 55, 61, 66], and promoting staff self-care [1, 14, 21, 23, 24, 31, 34, 35].

#### Participant vulnerability and protection needs

Authors suggest that a protection framework ensures participant safety needs take priority over research [19, 21, 35]. Maintaining confidentiality is seen as essential to avoid increasing participant vulnerability. For example, participants may be at increased risk if they are perceived to gain disproportionately from involvement in the research, such as being preferentially heard, included above other groups, or treated more favourably [21, 28].

Researchers from varying disciplinary backgrounds differ in their definitions of vulnerability, as summarised in Table 2:

**Table 2:** Bioethical, social science and mental health definitions of vulnerability

*Bioethics*	• Vulnerable populations are more susceptible to abuse and require additional protections [19] • The “vulnerable” are those likely to be misled, mistreated or taken advantage of, which imposes duty on researchers and ethical review boards (ERB’s) to ensure protections are in place [46].
*Social Sciences*	• Vulnerability is conceptualised as group status: powerlessness and potential for exploitation, those who lack the power and / or resources to speak out and make voluntary choices [46]. • Requires attention to individual and social vulnerabilities [46]. • Factors that influence vulnerability include exposure to disaster, individual capacity to cope, and the potential for serious crisis to occur as a result of exposure [46]. • Awareness of how displacement status (e.g., refugee or IDP), may affect individual vulnerability [32].
*Mental Health*	• Vulnerability defined in opposition to resilience: from a biomedical perspective, populations are seen as inherently vulnerable to adverse mental health reactions following disaster; whereas from a social sciences perspective the focus is upon the interactions between individual and community levels which may give rise to vulnerabilities [46] • Assumptions of participant capacity and autonomy are unjustified in emergencies, requiring extra protections to avoid exploitation [51].

All definitions of vulnerability are subject to critique, such as that the term is too elastic [46] and that it can stereotype and stigmatise [19]. Conversely, whilst accepting that a focus on vulnerability can lead to paternalism, O’Mathuna [18] argues that this also stimulates awareness of human fragility and the need to ensure protection from harm.

Authors call for an individualised response to vulnerability [35], recognising that it may arise as a result of specific settings, circumstances, or individual capacities [46]. Therefore, attention is drawn to the way researchers define and operationalise vulnerability, and the potential consequences that conferring “vulnerable” status may have upon an individual’s or group’s autonomy and agency in a specific emergency.

Participants involved in MHPSS research may present with specific protection needs including severe mental disorders [65], suicidal ideation [54], and sexual exploitation and abuse [21, 35]. Wissow et al [65] identify specific protection needs for people with serious mental health problems in emergencies, including: minimising lapses in medication, recognising the impact of social and economic disruption such as curfews, and ensuring equity of treatment access that may require identifying and engaging those who are marginalised. In often rapidly changing emergency contexts, it is essential that participant wellbeing is monitored [18] to ensure protection needs are identified and managed [37].

Vulnerability and protection are intimately linked to informed consent, assessments of capacity, and the potential for research to lead to exploitation [21, 45]. However, the Hastings Centre [36] argue that whilst vulnerability and protection needs may make exploitation more likely, these are neither necessary nor sufficient for its occurrence in any context.

#### Accountability

Accountability is conceptualised as being answerable to funders and the community in which research is conducted [47], requiring that researchers manage competing priorities [54]. In all settings, accountability entails having in place the resources required to support research. For MHPSS research in emergencies, authors identify key considerations such as: access to specialist mental health services [26, 29, 31, 43, 61]; meeting protection needs [23, 44]; and minimising physical and emotional harm attributable to research [32]. It is recommended that a referral booklet of services [31, 43, 61] and procedures for responding to suicidal ideation [54] are in place prior to starting the research. For severe mental health problems, authors highlight the duty to conduct legal review of deprivation of liberty (e.g., for persons at risk of harm to themselves or to others) [65].

Accountability further requires that researchers enter emergencies mentally, physically and materially prepared [20], and that they are competent and ready to practice [43]. This requires capacity building [24] and supporting local research infrastructure [20, 30, 31, 42, 47].

Emphasis is placed on research teams and auxiliary staff being fairly selected through transparent procedures [55, 66]. When working in conflict settings, authors argue that it is unethical to involve inexperienced researchers [15]. However, Jacobsen and Landau [28] caution that field experience is not a guarantee against poor practice, and that researchers’ expectations must be aligned to “on the ground” realities [43]. Researchers’ understanding of local culture is emphasised as being of particular importance in emergencies [31, 56, 64]. Additionally, the impact of interpreter and researcher backgrounds is highlighted [21, 32], including religion, culture, and ability to access to the study site and population [54]. When researchers are hired from within the study community, it is important to consider potential impacts upon confidentiality and anonymity [54], and how local attachments may make it difficult to negotiate traditional hierarchies [66]. In international collaborations, the need to understand asymmetries is recognised, including the extent that ethical discourse and practice are institutionally and professionally embedded [22].

In emergencies authors recommend all research staff (including drivers and translators) be provided training in their role and in ethical codes of conduct [1, 14, 21, 23, 27, 39, 43, 51, 52, 55, 61]. Hunt [55] argues training should aim to build a culture of ethical analysis and discussion as a natural discourse in emergencies. Table 3 outlines other recommended specialist training related to MHPSS research in emergencies.

**Table 3:** Recommendations for specialist training related to MHPSS research in emergencies

• Cross-cultural competencies [21, 27], including for researchers partnering with existing organisation staff in research [41];
• Basic helping skills such as Psychological First Aid [76] [23, 27];
• Identifying those at risk or considered vulnerable [45];
• Knowledge of referral pathways and responding to participant distress, vulnerability, and protection needs [14, 35, 43, 45, 61]; including ongoing monitoring procedures [24];
• How to recognise, establish and maintain professional boundaries [14] and manage issues not directly related to study conduct [61];
• Mental health skills including recognising severe mental illness [65];
• Risk management [39];
• Safety covering emergency preparedness, field coordination practices, background to the emergency [52], social and psychological risks associated with working in emergencies [61], and self-care [14];
• Understanding and implementing confidentiality and anonymity procedures [1, 14, 35];
• Data management procedures and dissemination arrangements [14];
• Background to the research topic [14];
• Specialist training in any tools, instruments and documents, including interviewers engaging and developing rapport with respondents [14];
• Specialist training that recognises the role of interpreters as active producers of research findings [51], covering confidentiality [1, 32] and power relationships [32].

In addition to training prior to research, authors recommend field mentoring [52, 55] and post-study debriefing [54] to ensure ongoing accountability and ethical reflection upon the particularities of working in emergencies. Some authors recommend using case studies as a pedagogical tool to develop ethical standards [14, 58].

#### Researcher self-care

As in many settings, authors recognised the potential for researchers and participants to suffer physical and emotional harm from research involvement [14, 24, 35]. Accountability entails a duty to monitor and support researcher self-care, protecting against the possible negative effects of conducting research in difficult contexts and on potentially sensitive topics [1, 14, 21, 23, 24, 31, 34, 35].

Vicarious trauma [1] and counter transference [34] - including reactions such as stress, grief, anger, and over-involvement in participants lives - are risks for researchers, particularly in resource constrained environments such as emergencies. Allden et al [21] argue that strategies to manage these reactions are especially required in qualitative research where participants may reveal intimate aspects of their lives and where professional boundaries can be more porous.

To ensure researcher self-care Tankink [34] calls for supervision throughout the research process, including during data analysis and dissemination. Others [31, 43] recommend that researchers work in pairs, and that organisational strategies to avoid burnout such as time off and ongoing self-assessment of competency to practice are implemented. Extending this, Curry et al [24] recommend that research staff give informed consent that includes explicit reference to increased health, security and other risks staff in emergencies are exposed to.

#### Environmental, political and health safety

Working in emergencies is characterised as working “in extremis” [43] due to the potential threats to personal wellbeing and safety. Ensuring the environmental, political and health safety of researchers and auxiliary staff is highlighted by many authors [14, 21, 24, 28, 35, 39, 43, 61, 63], and demonstrates respect for persons [37]. This encompasses having in place measures including exit strategies and procedures for safety monitoring, and accounting for any associated costs [24, 35].

Due to the changing nature of emergencies it is recognised that safety procedures must be able to respond to changing security threats [21]. Researchers may be at risk of violent attacks if they are viewed as a route to resources [28]; when meeting the protection needs of participants [35, 63]; or in situations requiring they breach confidentiality - for example when reporting illegal activity [61].

### Neutrality

Findings reflect that in all settings neutrality requires that researchers remain aware of social and economic inequalities; inequity of healthcare access; and social characteristics such as age, gender, religion, and ethnicity [31]. This is achieved by maintaining principles of equity and impartiality [64] through non-discriminatory delivery of resources and services [63]. In conflict contexts research occurs within an intensely political environment [15], requiring special attention to maintaining neutrality [31, 47, 63]. These background considerations frame the implementation of ethical research [16], requiring active awareness of power imbalances that are augmented in emergencies and bring an increased potential to cause harm [20].

#### Access, exit strategies, gatekeepers, and power

Curry et al [24] draw attention to security and exit strategies for planned research, including the circumstances under which research would be suspended or terminated such as in an acute crisis [61].

Ethical access requires coordination with existing systems [23] or “reverse triage” that hands the local community control over who enters an emergency and for what purpose [20]. However, emergencies present asymmetries in knowledge and power between researchers and participants that require mitigation [64, 67]. These may include structural economic, political and power inequalities, as well as situational inequalities such as resource access or psychosocial status [22, 32, 35]. Authors highlight that these may influence people’s motivation to participate in research, and can affect research validity [21, 28].

In emergencies it is acknowledged that access to settings and participants are frequently negotiated via a “gatekeeper”. The benefits of this approach include help to navigate socio-cultural [20] and bureaucratic systems, including knowing where to gain research approvals [68]. Conversely, risks include potentially augmenting hierarchies through controlled access to research benefits [21, 28, 35, 51, 56], or creating actual or perceived research bias in conflict contexts if negotiating access to participants via warring factions [15].

It is important to remain critical of who “speaks for” [22, 42] or represents a community, and to avoid privileging the voice of those with power or to silence those without [1, 47]. For example, gatekeepers may undermine the expression of some voices [32], including those related to the sharing of traditional cultural practices [52] or support systems [21]: “Research necessarily involves making political choices about which voices to hear and whose knowledge counts” [1]. Aube [64] recognises the tension in resisting local gatekeepers due to the potential for expulsion from the setting, putting research and services in jeopardy. Finally, Bäärnhielm and Ekblad [33] reposition the concept of gatekeepers by asking whether researchers themselves are viewed as gatekeepers to services or support.

#### Coordination with organisations and researchers

Collaborative partnerships are defined as sharing responsibility in all research stages in a relationship founded upon mutual respect [38]. Redfield [66] suggests that trust between local and expatriate researchers can be built through a shared commitment to humanitarian ideals achieved through research. Del Ben et al [68] recognise that collaboration between researchers and services offers opportunities for research and clinical care objectives to be met simultaneously.

Allden et al [21] draw attention to power differences between international and local researchers, and between researchers, service providers and communities, operating beyond categories of local / expatriate [30] and that can impact upon the research encounter. Such disparities can lead to the imposition of outside approaches and silencing of local practices [21], and demands critical awareness of “white knowledge dominance” [30].

Authors emphasise coordinating research with emergency response [26], ensuring it is networked into safety procedures, the socio-political emergency context [52], and specific MHPSS mechanisms and services [31, 43]. This is recommended based on the view that coordination efforts help to identify existing resources to support successful research [20, 26, 29]. External, consultant-led studies may cause challenges to coordination by putting expatriate researchers in a position of power over service providers [41], and present potential difficulties in responding to substandard care by local service providers involved in the research [55]. To address these authors recommend coordinating with enduring institutions [47] and establishing shared professional standards prior to starting the research [64].

It is recognised that poor coordination can lead to research duplication [52] and undue burdens for participants [20]. Failure to share findings and co-learn can limit efforts to provide comprehensive MHPSS support. To address this, Allden et al propose an open-source system to track data collection and facilitate coordination [21].

#### Declaration of researcher interests

In all settings, the ethical responsibility to declare researcher interests – including financial, career, and organisational or personal gains - is emphasised [24], ideally avoiding all conflicts of interest [37]. Conflicts of interest specific to research in emergencies may occur when delivery organisations commission research, and researchers compromise the integrity of the study by looking for findings that the organisation want to hear [18, 41], or when research is led by an external consultant and tensions occur between respecting cultural norms and imposing cultural values [64].

It is recognised that researchers, participants, ethical review bodies and organisations partnering in research all bring their own interests [1, 52]. These can affect setting research agendas, particularly in the presence of a “powerful outsider” [22], and lead to differing views of research success [30].

#### Funding

It is acknowledged that the extent to which aid is tied to funder priorities [21] or normative goals [22] may constrain how research funds are spent [42], and whether research is viewed as the wielding of power by funders or a political tool of governments [52]. Funders may have ethical frameworks or review processes which must be adhered to, frequently with an individualistic bias that may conflict with local cultural norms [60]. Conversely, Zwi et al [1] argue that funders are in a position to stimulate new ethical standards and ways of working.

Specific funding considerations relevant to emergency research include the implications of entry and exit strategies (e.g., research suspension or termination) [24], and the question of who funds research benefits such as ongoing access to services or treatments [36]. In addition, funders are often ill-equipped to judge the ethical and scientific rigor of research [21, 22]. Some authors call for funding to learn lessons about how research is conducted; putting into practice corrective efforts to ensure accountability [56]; recognising the benefits of potentially time-consuming research such as participatory methods; and the importance of accessible dissemination for collective learning [1, 21].

### Purpose and benefit

A range of considerations relating to research purpose and benefit in emergencies are identified, such as: ensuring direct benefits to participating communities [21, 68], building long-term collaborations that deliver sustainable benefit [31, 51], and disseminating findings to the participating community [21, 29, 47, 51]. These raise contested imperatives of sustainability [22, 56] and “reasonable benefits” that have stimulated academic debate [36–38].

#### Sustainable benefit

There is consensus that research participants should benefit from their involvement [1, 21, 24, 35–38, 41, 68]. Benefits range from the micro-level of occupying time, providing a sense of being heard [21], and access to the fruits of research [36]; to more generalisable benefits in the future social value of research [38, 42] such as improving service delivery [49].

There are debates about the level and timing of benefits [36] including mechanisms to benefit from results unknown at the study outset [37, 38]. Due to doubts about ensuring the future social value of emergency research, it is argued that direct benefits must also be assured [20]. Curry et al [24] propose that research protocols identify for whom and when benefit will arise, and, where relevant, how deferred benefit is ethically justifiable.

Authors call for avoiding “fly in-fly out” research [31, 51] in addressing research purpose and benefit: for example, is it a one-off endeavour or part of sustained involvement with a community [22, 30, 64]? Brown et al [22] argue that research should promote solutions embedded into existing systems and not a parallel aid system, emphasising sustainability and avoiding skewing local economies and job markets.

#### Dissemination

It is agreed that the participating community should be provided with research findings in an accessible format [21, 29, 35, 54], recognising these are a public asset [47] and that communities have a right to this information [51]. It is recommended that research be disseminated to local communities and policy makers [30]; and internationally to policy and academic audiences [30, 63], and funders [63].

Key issues relating to ethical research dissemination include data ownership, and the format and means of dissemination [21, 58]. Authors identify specific considerations heightened in emergencies, including potential inadvertent disclosure [21] and political manipulation [42] or misuse [28] of results.

Therefore, literature emphasises that the researchers’ role is to collect and disseminate information in a timely [20], scientific, and ethically sound manner [1], using publically accessible forums [24]. Failure to deliver this in any setting is seen as a breach of trust and the privileged relationship between researchers and participants [32, 51].

Difficulties predicting participant reactions to seeing oneself and one’s ideas described and objectified as symbolic and material resources are recognised [22]. These are seen to be heightened in LMIC settings, requiring efforts to ensure participants understand the implications of dissemination [21]. Hoeyer et al [60] argue that data should be shared with participants prior to dissemination, however challenges to this in emergencies are recognised, in particular population transience [21]. In emergencies it is important to remain aware of potential social, political or economic impacts that research interpretation and dissemination may have such as not reifying stereotypes, contributing to learned helplessness, or impacting upon the political will to aid those in need [41]. Brown et al [22] caution that research which aims to “give voice” can silence or downgrade other experiences, thereby causing harm.

Dissemination is recommended to include sharing data collection tools, methods [49], and results, including those that identify potentially harmful practices [69]. Dissemination should reach relevant audiences, recognising the importance of inter-agency learning [1] and ensuring research is not unnecessarily duplicated [14]. Sumathipala and Siribaddana [67] argue that journals should require evidence of local ethical approval and copies of informed consent to verify that overt exploitation has not occurred.

#### Ethical review

Authors agree that review by an institutional review board, ethical review board (ERB) or ethical review committee has become an accepted norm for research involving human participants [19, 63]. When research is well designed - including taking reasonable steps to protect participants – it is argued that it is *unethical* to prevent its conduct as findings should answer important questions to inform emergency response [18, 25]. ERB responsibilities include:

- protection of participants, particularly potentially vulnerable participants [38, 46];
- ensuring exploitation – inadvertent or intended – is avoided [24];
- verifying researcher training needs are identified and met [22, 24];
- providing public accountability [37] which includes educating and assisting researchers and communities in understanding research ethics, and ongoing research oversight - including data safety and monitoring [24];
- ensuring researcher transparency and accountability [38].

Authors critique ERBs for an inability to judge research conducted in emergencies [1, 21, 22, 31]. They argue that generic ERB processes offer little guidance or oversight [31] due to their lack of specificity to emergencies [22], which can lead to paternalism [1].

Some authors contend that ERBs consider their task in more legal than ethical terms [1] which can result in researchers having to persuade ERBs of the ethical imperative for research and the strength of strategies to mitigate risk when working with groups perceived “high risk” [39]. Awareness of the agendas of those conducting review, particularly in conflict or partisan contexts, is identified [22]. Emanuel et al [38] emphasise that researchers should seek to understand disagreement between different ERB judgements because this often relates to the relative weight of ethical principles by different bodies, whilst cautioning that the ethical standards of sponsor countries frequently prevail, potentially compromising participatory approaches towards protocol development.

## Consensus and unresolved debates

This section focuses on the distinctive features of applying ethical principles to MPHSS research conducted in emergencies, identifying areas where there is consensus and where there is disagreement. These were identified through the process of data analysis in which key tensions in the literature emerged either as points of discussion across papers, or through differing recommendations for managing or resolving key ethical tensions. The purpose of this section is not to offer an exhaustive discussion of these tensions, but to highlight where and why these controversies arise. This analysis of critical pressure points may be helpful to researchers reflecting on whether their research practice meets ethical standards identified as important for MHPSS research in emergencies.

At their foundation, ethical principles applicable to mental health research in emergencies correlate with universal standards [4, 5, 7, 8]. There is consensus over the relevance of universal ethical research standards to MHPSS research in emergencies, for example the accepted norms of ensuring participant informed consent; the importance of researcher neutrality, accountability and safety; and the imperative to ensure research is well designed and takes into account the contextual factors in specific emergency settings.

Beyond this consensus, it is in the application of ethical principles to MHPSS research in emergencies unresolved debates have been identified. The following discussion focuses on six debates with distinctive features in emergency MHPSS research, outlined in Table 4. Each is briefly discussed in turn, before drawing conclusions that point to an underlying tension between procedural and in-practice ethics [70].

**Table 4:** Unresolved debates

Issue:	
➢ What constitutes fair benefits? ➢ How should informed consent be operationalised? ➢ Is there a role for decision making capacity (DMC) assessments? ➢ How do approaches to risk management impact upon the construction of ethical research? ➢ How can ethical reflection best be achieved? ➢ Are ethical review boards (ERB’s) equipped to judge the ethical and scientific merit of emergency MHPSS research?	

### What constitutes fair research benefits?

Fair benefits for research participation has been extensively debated [36] and remains contentious for research conducted in LMICs generally, and emergency settings specifically [38]. There is consensus that there is an ethical imperative to maximise research benefits [19, 35, 37], and that benefits should be identified in direct relation to burdens [36]. However, how this could be implemented remains unclear beyond conducting a community assessment to verify that benefits / burdens are considered fair in a given context. Unresolved debates include questioning why the benefit of access to services is prioritised [36, 37] suggesting this confuses research with clinical care [38]. It is argued that other benefits could be of equal moral value, for example lasting policy and service impact [30] such as capacity building [31, 64] which increases the social value of research [38] beyond the “temporary” nature of emergencies [71].

The Hastings Centre [36] trace the fair benefits principle to the “reasonable availability” principle in the Council for International Organisation of Medical Sciences International Guidelines for Biomedical Research involving Human Subjects, charging that it fails to distinguish considerations including: what amounts to fair benefits - continued access to services, capacity or infrastructure building? To whom should benefits extend - participants, communities, an entire country? And who is responsible for funding benefits? Based upon these considerations, they conclude that the reasonable availability principle guarantees benefits but not necessarily *fair* benefits, and as such fails to protect against exploitation [36]. To remedy this a number of authors argue that researchers have a moral duty to clearly define research benefits, allowing participants to make an assessment of their fairness relative to burdens specific to the context and research topic [36–38], avoiding paternalism and ensuring respect for those in whose interest the research is conducted [40]. This approach accounts for each emergency having its own background structural and situational context including: the strength of existing MHPSS services; population exposure to experiences that may impact upon mental health; limited resources; and community identification of benefits of value to them.

### How should informed consent be operationalised?

There is consensus that it is the right of participants to be fully informed about research, and to voluntarily affirm their participation through providing informed consent. However, some authors contest the moral foundations of the informed consent concept [60], questioning whether consent protects participants or researchers [58], implying researchers serve self-interest in meeting quasi-legal rather than moral standards [37]. To achieve informed consent there are calls for moving away from procedural, juridical and ritualised consent, avoiding “a crude version of the biomedical model of consent: the dialogue should not be seen as merely … making the informant understand and accept a pre-defined research package” [59 - p.1746].

This is elaborated with a focus on the changing nature of emergencies, redefining the consent process to respond to evolving research [33], changing contexts [24, 52], or new information [38]. Additional considerations for consent likely to arise in LMIC emergency settings with largely collectivist cultures have been highlighted [19, 21, 22, 40]. These critique the individualistic bias inherent to informed consent, including a failure to acknowledge collective decision-making practices prevalent in some cultures [1]. Attention has also been raised to the potential inflexibility of funders and ERBs when it comes to what informed consent must “look like” [60]. Therefore, this review has identified tensions in how the ethical principle of informed consent is operationalised and implemented, with calls for prioritising cultural context and attainment of moral duties over quasi-legal standards through a more flexible and nuanced approach in practice [14, 21, 38, 52, 57], for example by approving a consent framework [51].

### Is there a role for decision making capacity (DMC) assessments?

Debates about the role of DMC assessments similarly reflect a tension between in-practice moral duties and procedural processes. There is agreement that respecting participant autonomy remains paramount in emergencies, understood as the ability to determine the direction of one’s life, make considered choices and act in accordance with one’s self-belief [51]. To uphold this and avoid harm it is argued requires assessments of DMC [18, 45]. However, there are differing views on the effect emergencies have upon DMC. These include assumptions of autonomy not holding [51, 52]; full autonomy being assumed unless reasonable reasons exist to think otherwise, drawing an analogy between the impact of exposure to emergency and having a severe mental health problem upon DMC [45]; or taking a middle road where DMC is seen as more severely affected in the acute emergency phase, thus requiring higher protections when research is conducted in this period [19]. Zwi et al [1] also note that participants may be motivated by fear, desperation or unrealistic expectations of assistance which may compromise DMC.

A wider moral concern relating to DMC assessments following emergencies is the potential to reinforce perceptions that mental health problems arises from exposure to emergency [45]. There are calls for proportionate procedures in which DMC safeguards are relative to the risk of harm a study presents [18, 45]. This debate remains unresolved as the proportionality of measures is based upon underlying assumptions of risk of harm, informed by an a priori understanding of the impact of emergencies upon capacity. Therefore, whilst there is underlying consensus about the moral duty of researchers to ensure trained research and clinical staff are able to identify and respond to participant protection and vulnerability needs – including impaired DMC - there remains debate about the assumed impact exposure to emergencies has on capacity.

### How do approaches to risk management impact upon the construction of ethical research?

As presented, the risks inherent to emergencies are understood to warrant higher protection of participants. However, there is considerable differences in the way that “inherent risks” are constructed and understood, and therefore what the appropriate response to such risks may be - illustrated by debates around DMC and the vulnerability of those exposed to emergencies [19, 32, 46, 51].

When considering the definition of risk in the Protection of Human Subjects, the US Department of Health and Human Services Code of Federal Regulations, states: “the probability and magnitude of harm or discomfort anticipated in the research are not greater in and of themselves than those ordinarily encountered in daily life” [72]. Iltis et al [39] highlight that in emergencies the “harms and discomforts” encountered in “daily life” automatically expose participants to higher risk. In light of consensus that populations perceived high risk deserve scientifically rigorous study [18, 25, 39], there is a call to action for researchers, funders and ERBs to develop and share innovative ways to manage risks inherent to MHPSS research in emergencies. This call addresses concerns that attempts to ensure ethical research can lead to protectionism, paternalism and a priori exclusion [19, 39], rather than a positive moral obligation to ensure those experiencing emergencies are afforded the right to evidence-based and ethical research and services.

### How can ethical reflection best be achieved?

One proposal for enhancing ethical research conduct is active reflection upon implementing ethical principles with a view to refining ethical research practice in specific contexts, and building transferrable knowledge for application across settings. Ways identified to achieve ethical reflection include study monitoring [37], conducting a post-study ethical audit following a structured checklist and involving all members of the research team [54], developing case studies based upon research experiences [14], and engaging in self-reflection [31]. Ethical reflection is argued for on the basis that it will support identification of best-practice [1] and, over time, development of practices for the application of ethical principles to emergency MHPSS research that account for contextual particularities conducting research in such settings give rise to.

Conducting ethical reflection complements the above discussions, promoting interrogation of research practice through an ethical lens with a view to enhancing the ethical foundation of emergency MHPSS research [11]. Such an approach recognises that ethical research necessitates a thoughtful process of balancing ethical considerations by researchers that should be rendered explicit [73]. Adopting a focus upon the specific needs of emergency-affected communities is foregrounds a concern for global justice [18] and promotion of a civic conversation around ethical research in emergencies [1].

### Are ERB’s sufficiently representative and equipped to judge the ethical and scientific merit of emergency MHPSS research?

International ethical guidance and review processes are charged with lacking focus upon the specific challenges that arise in emergencies [40]. Termed a “double-bind”, ERBs are able recognise risk and potential exploitation, but unable to offer practical guidance to address these [1 - p.266]. Procedurally it is recognised that in LMICs ERBs may be lacking or dysfunctional [74] with varying levels of expertise and professionalism to uphold ethical principles [75]. Due to the multiple levels of review, researchers frequently strike a compromise that draws upon sponsor country ERBs familiarity with research with vulnerable participants and where possible emergency settings, alongside engaging in-country bodies to certify that cultural norms and participants interests are adequately reflected [24]. Recognising these multiple layers of review, Curry et al [24] encourage researchers to identify the ethical review processes and bodies that will be involved in protocol approval, including known strengths, weaknesses, and ability to provide initial and ongoing ethical oversight.

Suggestions for overcoming these difficulties include: review by peers [49]; a bioethics service [45]; an ethical, social and cultural research ethics service [48]; or community-based advisory boards [21, 38]; and fast track processes [21] involving protocol pre-approval with adaptation to a specific emergency before final approval and study commencement [18]. For complex and evolving research such as ethnography, iterative ERB processes have been suggested [1]. These proposals aim to fill gaps in existing ethical procedures by addressing the asymmetries of in-country and international guidance, and to encourage co-learning between ERBs, researchers and communities.

An additional consideration is the extent that ethical review mechanisms established by bodies such as funders are equipped with technical and ethical expertise, and sufficiently divorced from normative priorities of donors, to provide independent review [21, 22]. Conversely, others argue that funders can stimulate new ethical standards and ways of working [1]. One proposal for clarifying the extent that funders promote or limit ethical research is to reflect upon potential discrepancies in different levels of review, providing opportunities for understanding how differences relate to the ways ethical principles are balanced, providing valuable contextual knowledge [38]. As this discussion summarises, current ERB procedures are not viewed as sufficiently responsive to the needs of emergency MHPSS research. However, there remains a lack of consensus about the ways to address and overcome shortcomings.

## Strengths and limitations

Due to the multidisciplinary and expansive approach of this review the procedures do not adhere strictly to those of a formal systematic review. There is little consensus on how the quality of qualitative research should be assessed [13], and in this review no quality assessment was undertaken. However, the majority of sources included in this review were published in peer-review journals or as peer-reviewed guidelines meaning that there was some level of quality check prior to inclusion. This broad inclusion criteria reflects the aim of the review: to identify sources that identify and discuss ethical principles applied to MHPSS research conducted in emergencies.

As the purpose of this review is to explore a range of perspectives – academic and practitioner - and to identify areas of consensus and debate relating to the ethical conduct of MHPSS research in emergencies, these limitations are deemed both acceptable and necessary for the present exercise. As a unique contribution and the first known attempt at systematically reviewing evidence on the application of ethical principles to MHPSS research in emergencies, this review fills an important gap in existing knowledge. Furthermore, the insights from this review have been applied to the development of evidence-informed recommendations for the ethical conduct of MHPSS research in emergencies [12], resulting in “real life” outputs.

## Summary and recommendations

This review of multidisciplinary literature has identified and discussed evidence on ethical principles applicable to conducting MHPSS research with adults in emergencies. Through searches on academic and practice databases applying broad inclusion criteria, 59 sources were identified and reviewed.

Discussion has revealed a systemic tension between procedural ethics and ethics in practice [70]. For some this is an attempt to straightjacket ethics [60], forcing complex social realities into procedures where the attainment of moral responsibility can end up playing second fiddle to quasi-legal standards [37]. Behind many of the debates raised is the desire to ensure the protection of participants exposed to emergencies and in need of evidence-based MHPSS services. However, there is a lack of consensus on how to achieve ethical research practice. A recent proposal for balancing the strict procedural “one-size-fits-all” against a relative approach that lacks common underlying normative foundations is to adopt a situated approach that prioritises contextual interpretation of ethical principles prior to their application [11]. This approach recognises the uniqueness of each emergency context and each research encounter, with active and continual consideration of the application ethical principles essential to ensuring research protects and promotes the rights of participants whilst making valuable contributions to the evidence base. This overarching consideration requires attention to direct future efforts to strengthen the ethical foundations of emergency MHPSS research.

## Conclusion

This review fills an important gap in knowledge relating to the ethical conduct of MHPSS research, identifying some key current debates. Through a broad literature review, we have sought to provide an overview of academic and field perspectives on the applicability and operationalisation of ethical principles when conducting MHPSS research in emergencies. This has been presented through the lens of five themes under which a number of ethical considerations have been identified, and their cross-cutting and mutually dependent nature demonstrated. These findings are important for understanding how the ethical challenges inherent to the conduct of MHPSS research in emergencies are responded to, identifying consensus approaches to achieving ethical research conduct in emergency settings.

A central principle underpinning the reviewed literature is a desire to ensure the protection of participants exposed to emergencies and in need of evidence-based MHPSS. However, there is a lack of agreement on how to contextualise guidelines and procedures to effectively maximise the perspectives of researchers, participants and ethical review boards. This is a tension that the field must address to strengthen ethical MHPSS research in emergencies.

It is hoped that this exercise will encourage further documentation of research experiences from an ethical perspective, continuing to build evidence about appropriate procedures and practice to inform how ethical principles are interpreted and applied in a challenging research field.

## Abbreviations

ERB: Ethical Review Board
DMC: Decision-making capacity
IASC-RG: Inter Agency Standing Committee Reference Group on Mental Health and Psychosocial Support in Emergencies
LMIC: Low and middle income country
MHPSS: Mental health and psychosocial support

## Footnotes

1 New IASC-RG membership is restricted to those organisations working in a minimum of 2 countries. For the development of the ethical guidelines that arose out of this review we were able to include the perspectives of national NGO’s.
