# Erratum to: Ethical standards for mental health and psychosocial support research in emergencies: review of literature and current debates

**DOI:** 10.1186/s12992-017-0264-2

**Published:** 2017-06-29

**Authors:** Anna Chiumento, Atif Rahman, Lucy Frith, Leslie Snider, Wietse A. Tol

**Affiliations:** 1 University of Liverpool, Institute of Psychology, Health and Society, 2nd Floor, Block B, Waterhouse Building, 1-5 Brownlow Street, L69 3GL, Liverpool, UK; 2 Independent Consultant, Peace in Practice, Amsterdam, The Netherlands; 3 Johns Hopkins University, School of Public Health and The Peter C. Alderman Foundation, Baltimore, USA

## Erratum

In the original publication [1] of this article on 8 February 2017 there are 3 errors related to the citations and their corresponding references. In this Erratum the correct and incorrect citations are displayed in **bold**. The page numbers for these citations were correct in the original publication. The citations in the original publication of this article have been updated.

The first incorrect reference & citation was in the below section:

1. Consent as “informed” is defined universally as: “an understanding of study purpose, who are the targeted beneficiaries, and the implications of involvement…information is communicated in a form appropriate to the culture, age, and educational level of that individual” **[14 - p.s224].**

- The above citation originally referred to: **[14] World Health Organisation. WHO Ethical and Safety Recommendations for Researching, Documenting and Monitoring Sexual Violence in Emergencies. Geneva: WHO; 2007. p. 1–33.**

The correct citation belonging to this sentence is:

- **[21] Allden K, et al. Mental health and psychosocial support in crisis and conflict: report of the Mental Health Working Group. Prehospital And Disaster Medicine. 2009;24**(**Suppl 2):s217–27.**

The second incorrect reference & citation was in the below section:

2. Despite the challenges, the researcher’s duty to safeguard privacy and confidentiality both during and after research is highlighted [15, 18, 27]: “anyone asking someone to disclose information bears a responsibility to safeguard that information**” [18 - p.18].**

b. The above citation originally referred to: **[18] O’Mathuna DP. Conducting research in the aftermath of disasters: ethical considerations. J Evid Based Med. 2010;3(2):65–75.**

The correct citation belonging to this sentence is:

- **[14] World Health Organisation. WHO Ethical and Safety Recommendations for Researching, Documenting and Monitoring Sexual Violence in Emergencies. Geneva: WHO; 2007. p. 1–33.**

The third incorrect reference & citation was in the below section:

3. To achieve informed consent there are calls for moving away from procedural, juridical and ritualised consent, avoiding “a crude version of the biomedical model of consent: The dialogue should not be seen as merely … making the informant understand and accept a pre-defined research package” **[59 - p.1746].**

c. The above citation originally referred to: **[59] Yamout R, Jabbour S. Complexities of research during war: lessons from a survey conducted during the summer 2006 war in Lebanon. Public Health Ethics. 2010;3(3):293–300.**

The correct citation belonging to this sentence is:

- **[60] Hoeyer K, Dahlager L, Lynoe N. Conflicting notions of research ethics. The mutually challenging traditions of social scientists and medical researchers. Soc Sci Med. 2005;61(8):1741–9.**
